# German Real-World Experience of Patients with Diverse Features of Acute Intermittent Porphyria Treated with Givosiran

**DOI:** 10.3390/jcm13226779

**Published:** 2024-11-11

**Authors:** Ilja Kubisch, Nils Wohmann, Thaddäus Till Wissniowski, Thomas Stauch, Lucienne Oettel, Eva Diehl-Wiesenecker, Rajan Somasundaram, Ulrich Stölzel

**Affiliations:** 1 Porphyria Center, Chemnitz Hospital, 09116 Chemnitz, Germany; i.kubisch@skc.de (I.K.); n.wohmann@skc.de (N.W.); t.wissniowski@skc.de (T.T.W.); l.oettel@skc.de (L.O.); 2 MVZ Labor Volkmann, 76131 Karlsruhe, Germany; t.stauch@laborvolkmann.de; 3 Department of Emergency Medicine and Porphyria Clinic, Charité—Universitätsmedizin Berlin, Corporate Member of Freie Universität Berlin and Humboldt-Universität zu Berlin, 10115 Berlin, Germany; eva.diehl-wiesenecker@charite.de (E.D.-W.); rajan.somasundaram@charite.de (R.S.)

**Keywords:** AIP, AHP, givosiran, siRNA, ALAS1, Ipnet, HR-QoL, chronic symptoms, sporadic attack, ALA, PBG, fatigue, Fatigue Assessment Scale (FAS)

## Abstract

**Background/Objectives**: Acute intermittent porphyria (AIP) is a metabolic disease characterised by neurovisceral crises with episodes of acute abdominal pain alongside life-altering, and often hidden, chronic symptoms. The elimination of precipitating factors, hemin therapy, and pain relief are strategies used to treat porphyria symptoms, but are often reserved for patients suffering recurrent, acute attacks. Givosiran (siRNA) is an emerging AIP therapy capable of silencing delta-aminolevulinic acid synthase-1 (ALAS1) and, in turn, reducing the accumulation of delta-aminolevulinic acid (ALA) and porphobilinogen (PBG) that precede porphyria symptoms. The aim of this study was to investigate the efficacy and safety of givosiran administration in patients with both acute and chronic AIP burden, who were poorly responsive to current therapies, using a personalised medicine approach. **Methods**: Real-world data were collected in consecutive patients treated with givosiran at an accredited German Porphyria Clinical Center. Biochemical, clinical, and HR-QoL outcomes were monitored alongside adverse events (AEs). **Results**: Twenty-eight patients treated between 2018 and 2024 were sub-categorised into groups corresponding to Ipnet terms 13 ‘Sporadic Attacks, 5 ‘Symptomatic High Excretors’, 5 ‘Prophylactic Heme’, and 5 “Recurrent Attacks’. The mean time from diagnosis to treatment was 9.2 years (range in months 1–324), and the mean duration of treatment was 30 months (range 3–68). After 6 months of monthly givosiran injection (2.5 mg/kg), all patients’ ALA levels reached <2ULN, and 60% of patients attained PBG levels < 2ULN (*p* < 0.001). These biochemical responses were not different between sub-groups (*p* > 0.05). Clinically, 75% of patients’ chronic and acute porphyria symptoms improved. The total patient populations’ annualised attack ratio (AAR) improved; Historical AAR: 2.9 (0–12.0) vs. Givo AAR: 0.45 (0–3.0) (*p* < 0.01). During follow-up, nine patients experienced minor breakthrough episodes. Of these, three patients required hemin infusion. An association between clinical success and a shorter interim period between diagnosis and treatment was evident (r = −0.522, *p* = 0.0061). All patients’ indices of HR-QoL improved under givosiran, including mental health (38%, *p* < 0.0001) and pain (38%, *p* < 0.0001). Patient-reported health (givosiran 77.9% vs. baseline 37.1%, *p* < 0.0001) and clinical outcome scores (86.9%: good–very good) were also positive. Two patients withdrew from treatment <6 months, citing fatigue, which was a common side effect. A mild elevation in liver enzymes (AST and/or ALT < 1.5ULN, 15.4%) and reduced glomerular filtration rates (GFR, 11.5%) were also evident, but no life-threatening adverse events (AEs) were attributed to givosiran treatment. **Conclusions**: Givosiran is effective in preventing severe acute attacks and reducing the chronic health burden in patients with acute intermittent porphyria. Importantly, HR-QoL improved in patients suffering chronic AIP burden with few incidences of historical attacks. All patients experienced substantially improved mental health, ease of living, and self-perceived health.

## 1. Introduction

Porphyrias are classified as either erythropoietic or hepatic, depending on the main site of intermediate accumulation along the heme synthesis pathway [1,2]. In acute hepatic porphyrias (AHP), the accumulation of neurotoxic precursors is considered a causal factor of neurovisceral crises, whereas a build-up of photosensitizing porphyrins is responsible for cutaneous symptoms in porphyrias that present with light sensitivity [3,4]. Among the hepatic forms (Acute Intermittent Porphyria: AIP, OMIM 176000; Hereditary Coproporphyria: HCP, OMIM 121300; and Variegate Porphyria: VP, OMIM 176200), AIP is the most common (comprising 80% of all symptomatic cases) [5]. It is autosomal dominant, with low phenotypical penetrance and a prevalence of 5–10 symptomatic cases per 1,000,000 [6,7]. AIP is characterised by a deficiency in hydroxymethylbilane synthase (HMBS), the enzyme responsible for the metabolism of porphobilinogen (PBG). AIP patients present with excessive levels of PBG and its precursor, delta-aminolevulinic acid (ALA) in the blood and urine [8,9,10]. Of these two porphyrin precursors, ALA is supposed to be neurotoxic, and emerging therapies are currently focused on disrupting the activity of aminolevulinate synthase 1 (ALAS1), the rate-limiting enzyme promoting its accumulation [11].

Most patients with AIP experience at least one attack and later become asymptomatic, requiring minimal clinical intervention. These patients report few incidences of porphyria attacks in their lifetime. Symptomatic cases can remain undiagnosed for an average of 15 years after disease onset [7]. Approximately 5% of patients with an overt form of the disease are chronically affected [6]. For these patients, limited treatment options exist that adequately target the disease or its symptoms, resulting in a markedly reduced health-related quality of life (HR-QoL).

Clinically, AIP is characterised by life-threatening acute neurovisceral episodes of severe abdominal pain, often accompanied by nausea, vomiting, tachycardia, hypertension, and hyponatremia [12]. These episodes are most often preceded by an identifiable trigger such as fasting, infection, porphyrinogenic medications, intake of xenobiotics, chronic alcohol consumption, stress, and the transient rise and fall of sex hormones that orchestrate the menstrual cycle, specifically, the luteal phase [13,14]. Given this, symptomatic AIP patients are predominantly female and of child-bearing age upon first presentation [6,15]. Attacks can be further complicated by neuropsychiatric features [16]. In the most severe cases, this can progress to hyponatremia, paresis, respiratory failure, coma, and death [17]. Long-term elevations of ALA and PBG are associated with other co-morbidities, including hypertension, end-stage kidney disease, chronic neuropathy, and primary liver cancer [1,7].

The diagnosis of overt AIP combines clinical evaluation and biochemical assessment with the possibility of further confirmatory enzymatic assays and genetic studies [18]. A diagnosis of AIP is suspected when increased concentrations of ALA and PBG are found in the urine, together with patient-reported symptoms. Since AIP is of low phenotypic penetrance, genetic testing is not a first-line diagnostic tool but can be used to identify family members potentially at risk of developing overt AIP [14].

Historically, drug therapy for AHP was limited to the treatment of acute attacks and was not designed to minimise chronic symptoms [19]. The first-line management of an acute attack includes pain relief with opioids and the elimination of triggers. Given that nausea and vomiting prevent carbohydrate consumption, intravenous (I.V.) glucose is routinely administered to reverse the fasting state [9]. Finally, this may be coupled with an infusion of hemin, the only specific treatment for porphyria designed to expand the intracellular regulatory heme pool, activating the feedback inhibition of ALAS1 activity and subsequently reducing the production of ALA and its metabolites [9]. Patients suffering recurrent acute porphyria attacks are commonly offered hemin infusion on a prophylactic basis every 1–4 weeks. Crucially, this treatment option is not curative and is associated with adverse effects such as venous obliteration, port infection, and iron overload [20,21]. Furthermore, frequent heme infusions may activate heme oxygenase 1, resulting in accelerated heme degradation and a loss of feedback inhibition of ALAS1 [22]. Patients with progressive disease, despite hemin administration, may only achieve complete symptom resolution following liver transplantation [23,24].

Recent clinical trials have demonstrated the potential value of a novel therapy for the prevention of recurrent attacks in AIP [25]. Givosiran is an ALAS1-directed small interfering RNA (siRNA) molecule first approved in 2019 by the U.S. Food and Drug Administration (FDA) for the treatment of adult patients with AIP [26]. Subsequently, it was approved in the E.U. in 2020 for the treatment of AHP in both adults and adolescents aged 12 years and older. Givosiran selectively silences the hepatic ALAS1 messenger RNA (mRNA), the rate-limiting enzyme driving the accumulation of ALA and PBG [27]. Conjugated to a trivalent N-acetylgalactosamine ligand, givosiran selectively binds to the asialoglycoprotein receptor preferentially expressed in the liver to enable targeted delivery to hepatocytes [28].

In patients suffering recurrent AIP attacks, data from the phase III ENVISION trial demonstrated givosiran’s ability to produce life-changing results, significantly reducing the annual rate of composite porphyria attacks, i.e., attacks requiring hospitalisation, urgent healthcare visits, or intravenous hemin administration at home [29]. Importantly, the investigators highlighted additional benefits of siRNA therapy, including a reduced reliance on hemin therapy and opioids [30]. However, patients experiencing chronic porphyria symptoms with fewer acute episodes did not meet the criteria for these initial trials (criteria: ≥2 attacks within the previous 6 months) and therefore, the evidence of a role for givosiran in the treatment of these patients is lacking. Further investigation is warranted to better understand the therapeutic potential of givosiran in patients exhibiting a spectrum of AIP symptoms with a marked impact on HR-QoL, representative of the wider symptomatic patient population.

The focus of this study was to assess the treatment outcomes and safety of givosiran, including in AIP patients with difficult-to-manage chronic porphyria symptoms that are poorly controlled by conventional treatment. Consecutive subjects receiving givosiran who attended a German Porphyria Clinical Center (PCC) were included.

## 2. Patients and Methods

### 2.1. Enrolment and Assessment

A real-world study was conducted on consecutive patients treated with givosiran between 2018 and 2024 at the Porphyria Centre, Chemnitz (PCC), an International Porphyria Network (Ipnet) accredited Porphyria Expert Clinical Center. Eligible patients were required to be ≥18 years of age with an AHP diagnosis confirmed by a specialist physician, evidence of urinary ALA and PBG levels ≥ 4 × ULN, and porphyria-induced symptoms. These symptomatic patients were subdivided into four distinct groups according to terms defined by Ipnet (Stein et al. 2023 [18]): “Sporadic attacks” (≤3 attacks within a 12-month period in the last 2 years)—Group 1; “Symptomatic High Excreters” (longstanding, chronic porphyria symptoms, without attack in the last 2 years, and a PBG/creatinine ratio ≥ 4 × ULN),—Group 2; “Prophylactic Heme” (received regular hemin infusion)—Group 3; and “Recurrent attacks” (≥4 attacks in 12 months, within the last 2 years)—Group 4. Following a referral from their local clinic, initial investigations detailed outpatients’ medical histories and assessed urinary ALA and PBG levels. Other parameters included liver enzyme activities (alanine transaminase [ALT] and aspartate transaminase [AST]), kidney function (estimated glomerular filtration rate, [eGFR]), lipase, and plasma homocysteine [Hcy]. Where possible, confirmatory genetic sequencing was performed to identify mutations within the hydroxymethylbilane synthase (HMBS) gene concurrent with AIP.

### 2.2. Dosing

**Figure 1:**
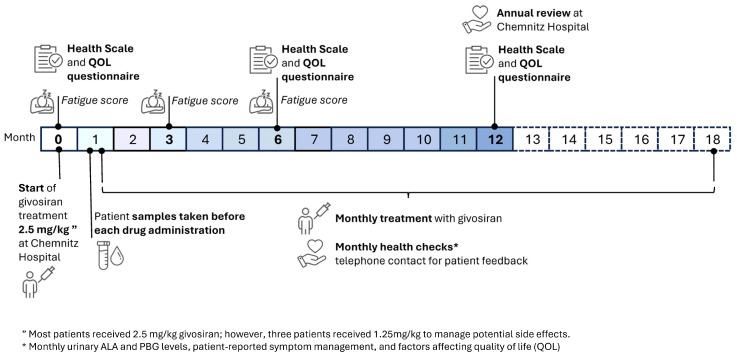
Overview of study timeline and design.

Givosiran therapy was initiated and managed by the PCC. The following monthly doses were mostly administered by a local general physician (GP), under remote management by the trial investigators. The initial dose was 2.5 mg/kg of lean body weight in 25 of 28 patients. A step-up dosing approach (initial dose 1.25 mg/kg) was agreed to for three patients who feared drug-induced side effects. Any acute porphyria attack during treatment was managed as per local standards of care [14]. A schematic overview of the dosing and data collection schedule is presented in the study timeline in Figure 1 below.

### 2.3. Data Collection

Prior to each drug administration, patient samples were taken for the follow-up assessment of urinary and blood parameters. Patients were required to complete a Health Scale, Fatigue-Scale, and QOL questionnaire on a bi-annual basis for at least one year.

For a measure of health state, we used the EuroQol group’s standardised EQ-5D-5L instrument previously employed to describe and value health across many disease areas. The EQ-5D-5L consists of the EQ visual analogue scale (EQ VAS), which ranks self-rated overall health from 0 (the worst health you can imagine) to 100 (the best health you can imagine) and a descriptive system evaluating five levels of severity in five dimensions of health (mobility, self-care, usual activities, pain/discomfort, and anxiety/depression) (Supplementary File S1). The level of fatigue was recorded using the Fatigue Assessment Scale (© FAS), a 10-item self-report scale evaluating symptoms of chronic fatigue developed for clinical research (Supplementary File S2). These, and other patient feedback opportunities, were facilitated by our clinical coordinator and one trial investigator via telephone, email, or online questionnaire. Patient management, data collection, and analysis were performed by trial investigators (IK, NW, and US) in accordance with the Declaration of Helsinki on Ethical Principles for Medical Research Involving Human Subjects, adopted by the General Assembly of the World Medical Association (2013). The investigation complied with the International Council for Harmonization Guideline for Good Clinical Practice (ICH-GCP) and all applicable national laws and regulations. Ethical approval was confirmed by the local ethical committee (2023). A schematic overview of the data collection is presented in the study timeline in Figure 1.

### 2.4. Objectives

Biochemical analysis of urinary ALA and PBG were expressed as absolute levels, relative to creatinine (Cr), or as a fold reduction relative to the baseline level. Biochemical response to givosiran was arbitrarily defined as <2ULN for ALA and PBG—for ALA: Upper Limit Normal (ULN) = 2.66 μmol/mmol of creatinine, 2ULN = 5.32 μmol/mmol of creatinine, 4ULN = 10.64 μmol/mmol of creatinine, and PBG: ULN = 1.01 μmol/mmol of creatinine, 2ULN = 2.02 μmol/mmol of creatinine, 4ULN = 4.04 μmol/mmol of creatinine.

Historical attacks were considered documented attacks before the application of givosiran and were defined by significant elevation of urinary ALA and PBG (>10ULN) and porphyria symptoms such as pain, autonomic neuropathy, and mental symptoms that required medical assistance and therapy (opioids, glucose, and/or heme treatment).

The Annualised Attack Rate (AAR) was calculated using the no. of attacks divided by time in months (between diagnosis and givosiran administration (historical), or time on givosiran, post-6 months (givo)), multiplied by 12.

We report the occurrence and severity of a ‘Breakthrough Attack’, defined by typical clinical features strictly in accordance with the Ipnet terms [18] whilst receiving concomitant monthly givosiran treatment (2.5 mg/kg of body weight). Givosiran strongly downregulates ALAS1 and the hepatic synthesis of porphyrin precursors. Therefore, PBG, as an Ipnet key biochemical criterion, could not be used for defining so-called breakthrough attacks. Clinical outcome, determined by the physicians’ overview of clinical success, was based on biochemical response (<2ULN), givosiran tolerance, and porphyria symptoms and was presented on a scale of 1–5 (1 = poor, 2 = unsatisfactory, 3 = satisfactory, 4 = good, and 5 = very good, respectively).

Post hoc analysis assessed factors affecting biochemical and clinical outcomes, including ‘time since diagnosis’, ‘historical heme infusion’, and the ‘duration of givosiran therapy’. Finally, follow-up interviews with patients assessed their perception of clinical success, expressed on a scale of 1–5 (1 = poor, 2 = unsatisfactory, 3 = satisfactory, 4 = good, and 5 = very good, respectively).

### 2.5. Statistical Analysis

The effect of givosiran-induced alterations on patients’ biochemistry levels, health, fatigue score, and HR-QoL over time was examined using *t*-tests (paired and unpaired) or one-way ANOVA (with Dunnett’s and Tukey’s test for multiple comparisons), where appropriate. Spearman’s rank correlation coefficient was applied to examine linear relationships between clinical outcomes and other factors. Data and statistical analysis were presented using GraphPad Software, Boston, MA, USA (v 10.2.3).

## 3. Results

### 3.1. General

#### Population

Between March 2018 and January 2024, a total of 28 patients with acute intermittent porphyria (AIP) underwent treatment with givosiran for an average duration of 30 months (range 3–68 months, Table 1). The patients were predominantly women (23 female and 5 male) with a mean age of 42.7 years (±14.8 years). Some patients had experienced a long period between a biochemically confirmed diagnosis and givosiran treatment, with the intermittent period between diagnosis and givosiran initiation ranging from 1 month to 27 years (or 324 months, mean 9.2 years, Table 1). Heterogenous HMBS mutations were confirmed in 95% of the patients sequenced (Supplementary Table S1).

Thirteen patients were characterised as having ‘Sporadic Attacks’—Group 1; five patients were ‘Symptomatic High Excreters’—Group 2; five patients were ‘Prophylactic Heme’—Group 3; and five patients were grouped as experiencing ‘Recurrent Attacks’—Group 4; (Table 1). Importantly, Groups 1–2 encompassed some patients who were excluded from other porphyria studies owing either to the sporadic nature of their attacks or their chronic symptoms (such as daily pain, significantly reducing QoL). Therefore, these patients’ responses to givosiran are, so far, underreported.

Prior to enrolment, the mean annualised attack rate (AAR) was 2.9 in the total patient population (*n* = 24 range 0–12.0), 3.02 for groups 1–2 (*n* = 16 range 0–12.0), and 2.65 for groups 3–4 (*n* = 8 range 0.5–5.14). Importantly, historical AAR was not significantly different between sub-groups 1–2 vs. 3–4 (*p* > 0.05, Figure 5). Historically, the management of prior attacks for all patients included acute I.V. glucose therapy, and most patients had received acute hemin infusion on at least one occasion (92.8%, Table 1). Six patients regularly received prophylactic hemin therapy that was initiated and managed by their local primary care institution (21.4%, Table 1). One of these patients was allocated to group 2 (Symptomatic High Excretor), given that their prophylactic heme therapy had been withdrawn many years before givosiran initiation. Another patient employed prophylactic gonadotropin-releasing hormone therapy to limit attacks precipitated by the menstrual cycle (3.6%, GnRH). Finally, more than a quarter of patients had been prescribed opioids for pain management between attacks (28.6%, Table 1).

### 3.2. Biochemical Response

#### 3.2.1. Three Months

**Figure 2:**
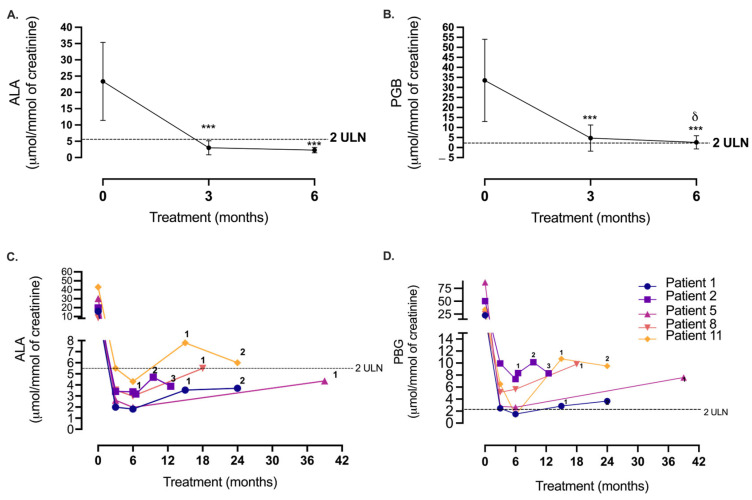
**Therapeutic siRNA givosiran alters the urinary biochemical profile of ALA and PBG in patients with AIP.** Urinary metabolites (**A**) delta-aminolevulinic acid (ALA) and (**B**) porphobilinogen (PBG) were measured following givosiran administration, presented as mean ± SD (μmol/mmol of creatinine) at 0 months (baseline), 3 months and 6 months treatment. Patients’ individual biochemical response, (**C**) ALA (*n* = 5), and (**D**) PBG (*n* = 5), following breakthrough porphyria symptoms (multiple events are numbered sequentially as 1, 2, and 3) under givosiran during follow-up (months). Dashed lines (----) represent biochemical thresholds (2ULN: ALA 5.32 μmol/mmol of creatinine, PBG 2.02 μmol/mmol of creatinine). Statistical significance was assessed via *t*-test (*** = *p* < 0.0001 (compared to baseline), δ = *p* < 0.05 (3 months vs. 6 months), *n* = 24–28).

Baseline measurements confirmed substantially elevated urinary ALA and PBG levels in all patients (mean ALA: 23.4 ± 12.01 μmol/mmol Cr (*n* = 28), and mean PBG: 33.5 ± 20.52 μmol/mmol Cr (*n* = 28)), concurrent with AIP. Following three monthly treatments of givosiran, a substantial decrease was evident in levels of both metabolites relative to baseline (*p* < 0.0001, Figure 2A,B). Here, 100% of patients’ ALA levels fell below <2ULN (<5.32 μmol/mmol Cr, Figure 2A). The PBG levels were also in significant decline (*p* < 0.001), reaching an average of 4.7 ± 6.5 μmol/mmol Cr, albeit this was still higher than four times the ULN for 32% of patients (>4.04 μmol/mmol Cr, Figure 2B).

#### 3.2.2. Six Months

At 6 months, we report a sustained reduction in PBG (relative to baseline, *p* < 0.001) and a significant further reduction in ALA between months 3 and 6 (3.0 ± 2.2 μmol/mmol Cr vs. 2.3 ± 0.8 μmol/mmol Cr, respectively (*p* < 0.05, Figure 2A,B)). In all patients, ALA fell below the <2ULN. Twenty-one (75%) of these displayed normal ALA levels (<2.66 μmol/mmol Cr, Figure 2A). In 17 patients (61%), the PBG- = level fell < 2ULN (<2.02 μmol/mmol Cr), with 8 of these patients (28%) presenting with normal PBG levels at 6 months. Notably, in 11 patients, PBG levels exceeded 4ULN (>4.04 μmol/mmol Cr) (Figure 2B) by 6 months.

#### 3.2.3. Patients’ Biochemical Response in Breakthrough Attacks

A total of nine patients on givosiran treatment reported symptoms corresponding to previous attacks, defined as breakthrough attacks. We were able to document five of these patients’ biochemical profiles following the attack, demonstrating increased urinary ALA and PBG concentration during the follow-up period (6–36 m). Notably, all five patients’ PBG levels exceeded four times the ULN following the first breakthrough attack (Figure 2C,D). Prior to experiencing their first breakthrough event under givosiran, two patients failed to demonstrate a reduction in PBG levels < 4ULN at 6 months (Patients 2 and 8, Figure 2D).

#### 3.2.4. Biochemical Response to Givosiran Across Ipnet Clinical Disease Classification

**Figure 3:**
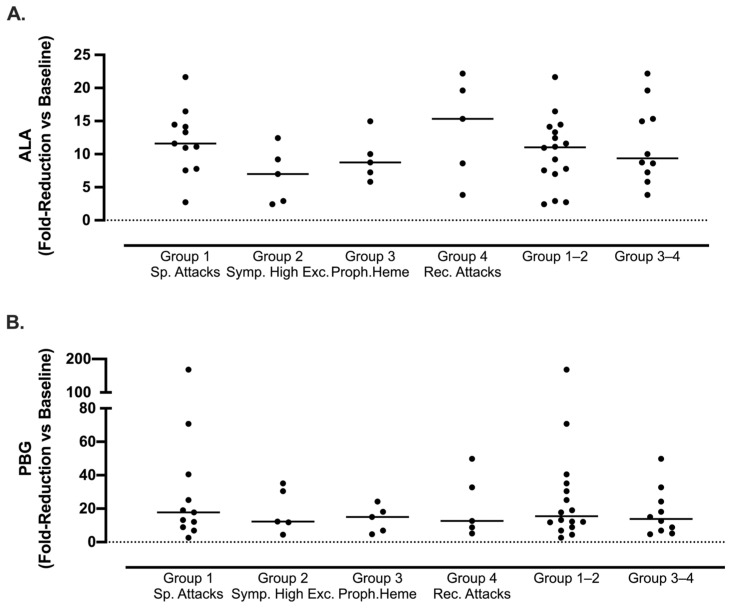
Givosiran treatment is similarly efficacious in its reduction in urinary metabolites ALA and PBG across phenotypically distinct AIP patients. Results after 6 months of givosiran treatment in 26 patients with AIP. Comparison of reduction (fold reduction) of urinary metabolites (**A**) delta-aminolevulinic acid (ALA) and (**B**) porphobilinogen (PBG) among phenotypically distinct AIP patients, divided into four groups according to Ipnet definitions. Group definitions: Group 1—Sporadic Attacks (*n* = 11), Group 2—Symptomatic High Excretor (*n* = 5), Group 3—Prophylactic Heme (*n* = 5), and Group 4—Recurrent Attacks (*n* = 5). Phenotypes are presented separately and collectively as Group 1–2 (*n* = 16) and 3–4 (*n* = 10) to demonstrate efficacy in patients (Group 1–2) who were excluded so far from givosiran treatment in other porphyria studies owing either to the sporadic nature of their attacks or chronic symptoms. Values are expressed as a fold reduction relative to baseline levels. Higher values indicate a higher reduction in ALA/PBG. Statistical significance was assessed via one-way ANOVA with Tukey’s multiple comparison test (*p* > 0.05). Two patients withdrew from givosiran at month 3 and are not included.

Patients across all AIP sub-groups demonstrated a similarly improved biochemical response to givosiran at 6 months, with a median fold reduction in ALA: 11.03 (range 2.4–21.6, Group 1–2) vs. 9.4 (range 3.8–22.2, Group 3–4, *p* > 0.05), and PBG: 15.5 (range 2.5–168.3, Group 1–2) vs. 13.8 (range 4.6–35.1, Group 3–4, *p* > 0.05), relative to baseline (Figure 3A,B). These improvements were not significantly different between groups (*p* > 0.05).

### 3.3. Clinical Response

#### 3.3.1. Clinical Outcome

**Figure 4:**
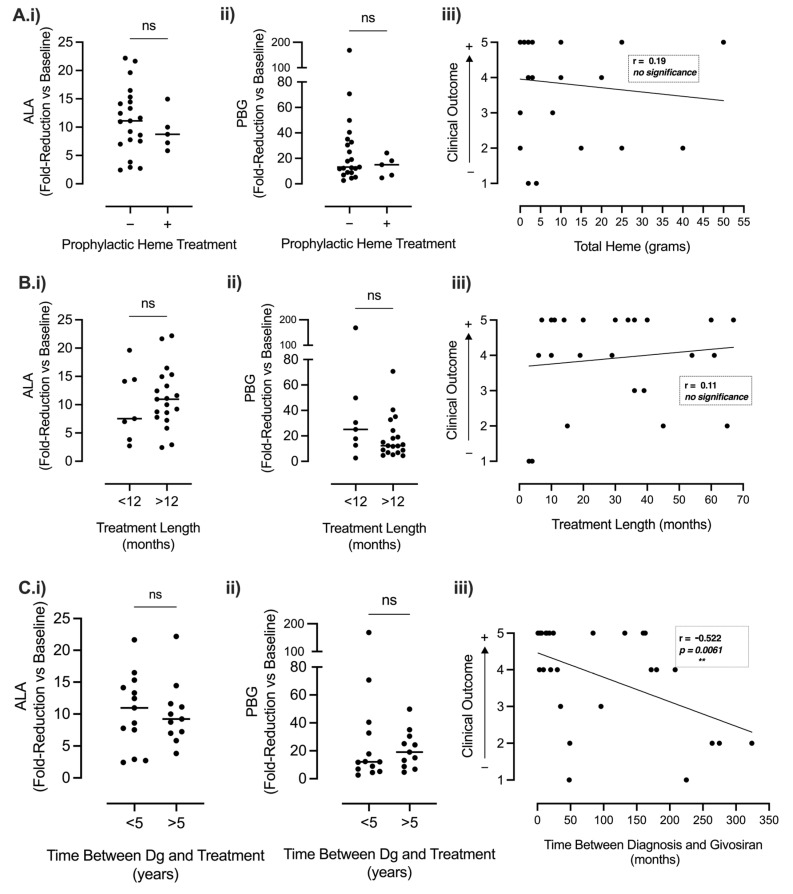
**Factors affecting the biochemical and clinical outcome of givosiran treatment in patients with AIP.** The effect of (**A**) prior prophylactic heme treatment (**i**,**ii**:—none, +regularly, or **iii**: the total amount historically infused in grammes), or (**B**) givosiran treatment length (**i**,**ii** <12 months or >12 months, or **iii**: 0–70 months), or (**C**) Time between AIP diagnosis and givosiran treatment (**i**,**ii** <5 years, >5 years, or **iii**: 0–350 months): on (**i**) ALA-level fold reduction at 6 months, (**ii**) PBG-level fold reduction at 6 months, or (**iii**) clinical outcome (measured on 1–5 scale, worst–best, respectively). For biochemical analysis, individual patient data point distribution is presented as a scatter plot with a median value, and statistical significance was assessed via unpaired, *t*-test, *p* > 0.05, *n* = 26. Simple linear regression with Spearman’s correlation coefficient represents the goodness of fit (r) and statistical significance (** = *p* < 0.01, *n* = 26), ns (not significant, *p* > 0.05).

Taken together, we demonstrate a positive clinical outcome (see definitions) in 75% of patients with givosiran reducing the incidence of chronic porphyria symptoms and acute breakthrough attacks in most (clinical success according to physician: mean score of 3.8 out of 5 (±1.3, Figure 4)). Two patients (7.1%) withdrew from the givosiran treatment after 3 months, principally citing overwhelming fatigue. A further three patients continued to suffer from chronic porphyria symptoms during givosiran therapy, despite two attaining an excellent biochemical response. One longstanding patient (treatment duration of 62 months) paused givosiran and reinitiated treatment at 50% dosage 6 months later, reporting decreased muscle cramps formerly compounded by givosiran-induced side effects. Moreover, a separate patient reported dysphagia, diagnostically confirmed as achalasia, that disappeared with givosiran cessation. Consequently, the reappearance of porphyria symptoms prompted the patient to re-initiate givosiran at 50–75% dosage, leading to more manageable dysphagia and a successful continuation of treatment.

**Figure 5:**
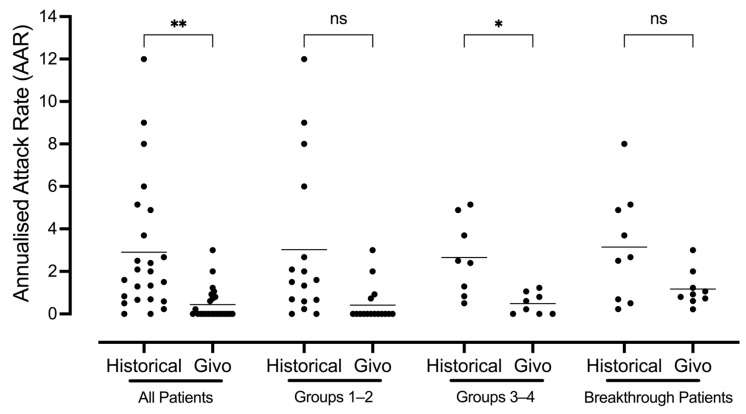
**Givosiran reduces the incidence of acute attacks in patients with distinct porphyria phenotypes.** The historical annualised attack rate (AAR) is compared with the AAR under givosiran after 6 months in patients with phenotypically distinct AIP patients divided into four groups according to Ipnet definitions. Group definitions ‘All patients’ (*n* = 24, based on the availability of historical data); Group 1—‘Sporadic Attacks’ (*n* = 11) and Group 2—‘Symptomatic High Excretor’ (*n* = 5), Group 3—‘Prophylactic Heme’ (*n* = 4), and Group 4—‘Recurrent Attacks’ (*n* = 4). Paradoxically, patients in Groups 1–2 display a high mean AAR due to a number of attacks in their total disease history (>24 months before givosiran initiation), regardless of their Ipnet classification exclusively employing a timeframe of 2 years. ‘Breakthrough Patients’ are the subset of patients experiencing porphyria attack symptoms under givosiran (“Givo”) after 6 months (*n* = 9). Statistical significance was assessed via a one-way ANOVA with Dunnett’s test for multiple comparisons ** = *p* < 0.01, * = *p* < 0.05 (mean historical AAR vs. mean Givo AAR), ns (not significant, *p* > 0.05).

In total, 96% of patients were attack-free during the first 6 months of givosiran treatment, and in the subsequent follow-up period, 65.3% of patients reported symptom relief. The mean AAR was significantly reduced within the total patient population, during follow-up: 0.45 (range 0–3.0), compared with historical AAR: 2.9 (range 0.0–12.0, *p* < 0.01, Figure 5). Moreover, in sub-groups 3–4, a paired analysis revealed that the AAR under givosiran treatment was also significantly lower than historical AAR (*p* < 0.05). A similar trend towards AAR reduction was also evident in Groups 1–2, but not statistically significant (*p* = 0.0503), as can be seen in Figure 5. These data suggest that givosiran can alleviate the recurrence of acute porphyria symptoms in patients with distinct AIP phenotypes, according to the Ipnet classifications.

Follow-up information available for 25 patients revealed that 36% (nine patients) had experienced at least one acute breakthrough attack during givosiran therapy. Only five patients experienced more than one acute attack; three of these five patients had received prophylactic hemin therapy on a regular basis prior to givosiran initiation (Prophylactic Heme, Group 3). Importantly, none of the breakthrough attacks were severe, and three of these patients presented to the hospital for I.V. glucose or heme administration. No patient required ICU treatment.

Moreover, the paired analysis revealed that in those patients who had experienced a breakthrough attack (36%), givosiran treatment did not significantly improve their AAR, despite a trend towards improvement (historical mean AAR 3.15 (range 0.23–8.0) vs. mean AAR under treatment: 1.18 (range 0.22–3.0), *p* > 0.05 (Figure 5)).

#### 3.3.2. Factors Affecting Clinical Outcome

In post hoc analyses, we report a negative linear association between a longer time between diagnosis to treatment and clinical outcome. Here, a shorter disease course between diagnosis and treatment was associated with a more favourable clinical outcome (R= −0.522, *p* = 0.0061, Figure 4C(iii)). However, a shorter disease course was not associated with a superior biochemical response to givosiran (*p* > 0.05, Figure 4C(i,ii)). Neither historical hemin therapy nor givosiran treatment length had any significant bearing on patients’ biochemical response to givosiran either (*p* > 0.05, Figure 4A,B(i,ii)). Similarly, we report that neither of these factors was of consequence to the overall clinical outcome (historical hemin, R = 0.19, *p* > 0.05; treatment duration R = 0.11, *p* > 0.05, Figure 4A,B(iii)).

#### 3.3.3. Patient vs. Physician Perception of Clinical Outcome

**Figure 6:**
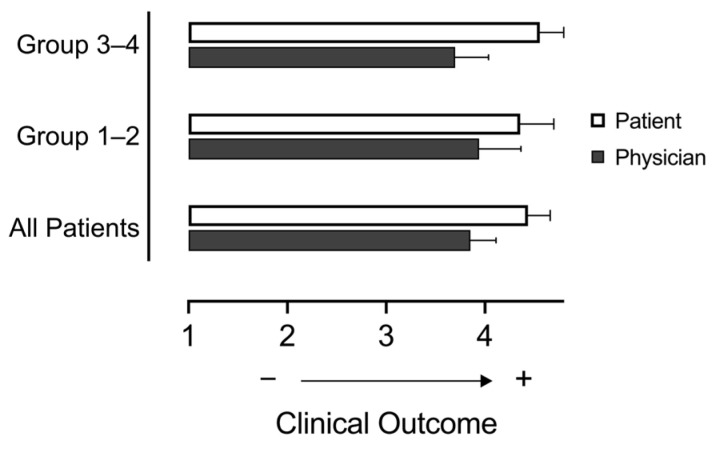
**Patient versus physicians’ perception of clinical success under givosiran.** Clinical outcome was measured on a patient- or physician-reported scale of 1–5 (1 = poor, 2 = unsatisfactory, 3 = satisfactory, 4 = good, 5 = very good). Data are presented together: all patients (*n* = 23) or as collective groups: 1–2 (*n* = 14), Group 3–4 (*n* = 9), versus physicians (*n* = 27). We assessed statistical significance via two-way ANOVA with multiple comparisons (*p* > 0.05).

We were able to ask 23 patients to rate their own clinical satisfaction. Most of these perceived their clinical outcome to be very good (69.5%) or good (17.4%). One patient rated their outcome as ‘satisfactory’ (4.3%) relaying one wish: “to be healthy”. Two patients rated their outcome less than satisfactory (8.6%). Largely, patients reported equally positive outcomes compared to their physician (patient: mean 4.43 ± 1.07 (good); vs. physician: mean 3.85 ± 1.35 (satisfactory-good), *p* > 0.05, Figure 6). A more conservative trend in physician opinion existed across all AIP sub-classifications, most prominently between patient vs. physician-perceived opinion in Groups 3–4 (*p* = 0.09). Consequently, it is possible that givosiran treatment provided this sub-group with a greater perception of clinical benefit, an effect underestimated by their physician.

#### 3.3.4. Quality of Life (QOL)

**Figure 7:**
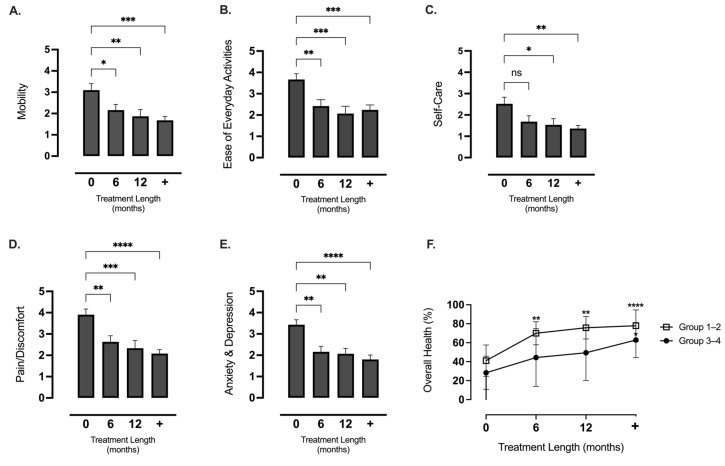
**Givosiran improved all aspects of the quality of life in AIP patients.** The effect of givosiran treatment on self-reported aspects of QoL in all patients (*n* = 18–25, some lost to follow-up), using the EQ-5D-5L Questionnaire measuring the following: (**A**) Agility, (**B**) Ease of everyday tasks, (**C**) Anxiety and depression, (**D**) Self-care, or (**E**) Pain/discomfort; expressed on a scale of 1–5 (1 = best, 5 = worst). (**F**) Overall Health Scale (patient-perceived wellness) is expressed as a percentage of optimal health (100%) in collective groups 1–2 vs. 3–4 (*n* = 9–16, *n* = 7–9, respectively); at baseline (0 months), 6 months, 12 months, and on final assessment (+). Statistical significance was assessed via one-way ANOVA, with Dunnett’s test for multiple comparisons comparing all measurements to baseline values (0 months) (* = *p* < 0.05, ** = *p* < 0.01, *** = *p* < 0.001, **** *p* < 0.0001), ns (not significant, *p* > 0.05).

We report that givosiran improved all aspects of patients’ HR-QoL, measured in the EQ-5D-5L questionnaire, and this was sustained with ongoing treatment (*p* < 0.001, Figure 7A–E). The scores reflected the greatest benefit of givosiran treatment on mental health (improved 38%, *p* < 0.0001), pain reduction (38%, *p* < 0.0001), and increased ease of everyday living (30%, *p* < 0.001, Figure 7A–E). Anecdotally, one patient stated that “being able to run really brings you back to life”, highlighting the importance of living life well during treatment for a chronic disease.

#### 3.3.5. Health Scale Index

We report that givosiran treatment produced a similarly marked improvement in patient-reported health, as measured by the Health Scale in the EQ-5D-5L questionnaire, across the phenotypically distinct AIP patient sub-groups: Groups 1–2: follow-up, givosiran: 77.9%, ±16.7 vs. baseline: 41.1%, ±16.6, (*p* < 0.0001, Figure 7F). Groups 3–4: follow-up, givosiran: 62.8%, ±18.5 vs. baseline: 28.3%, ±17.4, (*p* < 0.05, Figure 7F). A minor difference between collective sub-groups suggested that significant improvements to health were evident within a shorter treatment period for patients in Groups 1–2 (*p* < 0.01 at 6 months), compared to those in Groups 3–4 (*p* < 0.05 at >12 m+, Figure 7F). There were no significant differences between sub-groups at any time point (*p* > 0.05, Figure 7F)

### 3.4. Safety

#### 3.4.1. Side Effects

**Figure 8:**
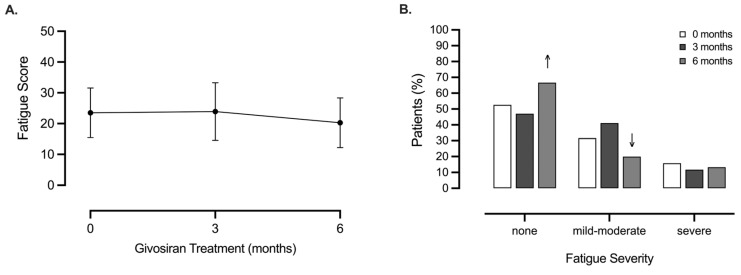
**Fatigue is variable in AIP patients undergoing givosiran treatment.** We measured (**A**) fatigue scores and categorised this by (**B**) severity (‘none’ <21, ‘mild–moderate’ >22–34, and ‘severe’ >35); expressed as fatigue score (**A**), or percentage of patients (%) (**B**), respectively. All measurements were assessed at baseline (0 months), 3 months, and 6 months under givosiran treatment (*n* = 15–19, some data lost to follow-up). Statistical significance was assessed using one-way ANOVA with repeated measures (*p* > 0.05), with arrows indicating a trend of improvement in fatigue at month 6.

Drug-induced side effects resulted in an unsatisfactory clinical outcome for six patients (21.4%). Two patients withdrew from the study, citing unacceptable fatigue following exposure to givosiran for 3–4 months. Givosiran-induced fatigue remained an individual and complex side effect amongst our patient population; in choosing to cease treatment, one patient affirmed that they would “prefer pain over fatigue”. Fatigue Assessment Scale scores demonstrated that many patients experienced moderate fatigue at 3 months (41%), but this was short-lived and has been mitigated after 6 months of treatment (20% of patients reported moderate fatigue, and 66% of patients reported no fatigue at 6 months, Figure 8A,B). Therefore, for some patients, this side effect may have important consequences for givosiran’s tolerability during treatment initiation.

Other commonly noted side effects of givosiran were muscular cramps (36%), nausea (29.1%, Table 2), and pain at the injection site. Three patients experiencing unsatisfactory side effects continued their treatment but reduced their monthly givosiran dosage to 50–75% to better manage this. Less commonly reported side effects included disturbed sleep, weight gain, drowsiness, and reduced performance.

#### Adverse Events

Adverse events (AEs) are also listed in Table 2. Renal AEs included an early but transient moderate increase in serum creatinine (30.7%) or a reduction in the eGFR (11.5%, Table 2). In total, 15.4% of patients experienced a mild elevation of liver enzyme activities (<1.5 UNL, [AST/ALT]), with a further 7.7% of patients’ liver enzyme activities elevated further (>1.5ULN, [AST/ALT]). There were no episodes of pancreatitis. An increased level of plasma homocysteine [Hcy] was evident in all patients under givosiran treatment (100%, Table 2). All patients were treated with vitamin B6 supplementation to mitigate Hcy levels.

One patient, previously on prophylactic heme, experienced a cerebral sinovenous thrombosis (Table 2). Notably, this patient had a significant history of smoking alongside additional pre-existing vascular co-morbidities. Given this, we did not attribute this AE to givosiran.

## 4. Discussion

There are few drugs available to treat acute intermittent porphyria (AIP), and historically, patients have endured chronic and worsening symptoms with little prospect of effective long-term treatment [13,31]. In the last 5 years, givosiran has transformed the landscape of AIP treatment via injectable mRNA silencing of the ALAS1 enzyme, in turn downregulating the production of potentially neurotoxic heme precursors ALA and PBG [32]. Given its ability to target the disease upstream, givosiran is an emerging therapeutic foremost prescribed to alleviate the burden suffered by AIP patients with acute, recurrent, neurovisceral crises, lessening the requirement for symptomatic therapies [21,29]. The ENVISION trials focused on investigating givosiran in this patient population, advancing into an open-label extension (OLE) period after 6 months [25]. Yet, many patients with AIP experience chronic symptoms with infrequent attacks, suffering a marked decline in HR-QoL whilst managing a slew of ‘hidden’ symptoms such as pain, nausea, tiredness, trouble sleeping, and anxiety [33,34,35]. Given this, studies further exploring givosiran therapy across a dynamic spectrum of AIP patients are warranted.

Here, we examined the therapeutic potential of givosiran in a patient cohort with different AIP phenotypes recently defined by Ipnet. Importantly, about half of our cohort (groups 1 and 2) would have been ineligible for the ENVISION trial, having either a limited number of attacks or lacking historical prophylactic heme treatment. Our results confirmed the ability of givosiran to drastically reduce both acute and chronic symptom severity across diverse patients, including those with designated ‘sporadic attacks’ and ‘symptomatic high excretor’ phenotypes (Stein et al., 2023 [18]). Using givosiran, we not only target the number of attacks but also the symptoms severely impairing the QoL in patients of AIP.

Importantly, this work investigated factors that affect clinical outcomes in closer detail than previous studies, resulting in an in-depth analysis that better reflects givosirans’ impact, reaching further than commonly reported ‘attack incidence’. This further translated to an improvement in HR-QoL for all patients across all indices, including mental health and chronic pain (*p* < 0.0001, Figure 6). Other studies employing a patient-partnership approach also report a sustained improvement in symptomatic patients alongside manageable tolerance levels [36]. Givosiran treatment could be paused in two female patients, providing them an opportunity to pursue pregnancy.

The biochemical repression of heme intermediates ALA and PBG was evident in patients of all AIP sub-types following 3 months of givosiran treatment, and this was sustained at 6 months. During follow-up, we evidenced that low precursor levels (100% of patients’ ALA levels were <2ULN at 6 months) were disrupted in a “flare” of breakthrough attacks occurring in 35% of patients. Still, these patients’ ALA and PBG levels remained reasonably low on these occasions with the driving precursor, ALA, exceeding twice the ULN in only two patients (Figure 2C). This is a strong indicator of givosiran’s biochemical efficacy, given that a patient’s biochemistry levels can reach more than 20–50 times the ULN during an acute attack [37]. Furthermore, breakthrough attacks under givosiran were minor, with only 33% of patients with acute symptoms requiring outpatient hospital treatment—a finding also reflected in the phase III ENIVISION trial [38].

In the overall patient population, the average AAR was significantly reduced under givosiran (*p* < 0.001), except for nine patients experiencing recurrent breakthrough attacks whose AAR remained unaffected (although there was a trend towards decrease). Notably, four out of nine patients were categorised as ‘prophylactic hemin’, suggesting that patients on prophylactic hemin may be predisposed to breakthrough attacks. However, due to a limited number of patients, this could not be evidenced in our study. Others report that historical prophylactic hemin had no impact on the givosiran-mediated reduction in the AAR [29]. Here, we further demonstrate that prior heme status had no bearing on the fold-reduction in ALA, or PBG, within the total patient population under givosiran, and likewise, we found no association between the amount of heme historically administered and the overall clinical success of givosiran (Figure 4A(iii)). Moreover, ‘prophylactic heme’ patients gained up to 40% improvement across all HR-QoL indices (Figure 7) and reported a positive overall clinical outcome in agreement with their physician (Figure 6) despite the occasional recurrence of breakthrough attacks under treatment.

Interestingly, patient optimism regarding clinical outcomes was common in our patient cohort, providing valuable insight into therapy outcomes that have been observed by others [39]. Critically, it also challenges the clinical endpoints utilised by clinicians to gauge the therapeutic success of a drug, potentially underestimating its overall reach. This was recently highlighted by authors exploring HR-QoL outcomes in RTC for cancer therapeutics [40]. We encourage discussion surrounding the inclusion of HR-QoL outcomes as a primary endpoint in RCTs investigating emerging experimental therapeutics, like givosiran.

As has been previously reported [38,41], patients with a longer disease course garnered a less successful clinical outcome under givosiran here. Given that the long-term consequences of residual chronic AIP symptoms lead to co-morbidities such as hypertension, increased risk for primary liver cancer, and chronic kidney disease, it is unsurprising that these issues are difficult to overcome at later stages of the disease [33]. Critically, this emphasises the need for early detection, diagnosis, and treatment of acute hepatic porphyrias. Recently updated Ipnet definitions, inclusive of patients with diverse AHP symptoms, represent a valuable resource for primary providers in early detection [18].

We can confirm that all patients in this study demonstrated an increase in total plasma homocysteine (Hcy). This is commonly reported in givosiran studies and is thought to be a result of ALAS1 silencing cystathionine beta-synthase activity [36,42]. We report one cardiovascular event in a patient under givosiran. Notably, the patient had pre-existing cardiovascular risk factors (smoking, obesity). Therefore, this was not attributed to givosiran or HHcy (hyper-homocysteine).

The ENVISION trial reported that givosiran is associated with several AEs, the most frequent being nausea, injection site reactions, fatigue, chronic kidney disease, and increased ALT levels [25]. Another Phase I study found common AEs to include nasopharyngitis, abdominal pain, and diarrhoea [43]. A recent review highlighted an increased risk of hepatic and renal adverse events [44]. In our investigation, the most frequently occurring AEs in decreasing order were HHcy (100%), muscular cramps (38%), fatigue (34%), impaired renal function (30.7%), and further decreasing eGFR levels (in patients with pre-established renal insufficiency) (11.5%), alongside a mild (15.4%, <1.5ULN) and moderate (7.7%, >1.5ULN) elevation of liver enzyme activities. These AEs are slightly lower than those reflected in the ENVISION study, whereby the safety profile of givosiran is considered acceptable [29]. Fatigue was a problem for some patients more than others, prompting two patients to entirely withdraw from treatment earlier than 6 months. Data from ENVISION cite fatigue in all patients, regardless of givosiran treatment [25]. However, we report more individualised and variable fatigue responses under treatment with improvement over time. Given that givosiran repression of ALAS1 expression induces a sharp dip in vital heme precursors, the subsequent shortfall in heme bioavailability may have repercussions for other iron intermediates within the liver, including enzymes essential for drug and endogenous compound metabolism, such as the Cytochrome P450 superfamily [32]. It could be suggested that downstream alterations in liver metabolites might acutely affect patients’ energy levels, becoming chiefly problematic during givosiran initiation. This could be of further consequence for patients taking other medications, impeding efficient drug metabolism and heightening the potential of drug interactions. Authors Vassilou et al. demonstrated moderate reductions in CYP1A2 and CYP2D6 under givosiran, which could have implications for the metabolism of caffeine-like and dextromethorphan-like compounds, respectively [42]. We hypothesise that as the heme pool slowly repletes, this shortfall may improve, accounting for early fatigue that subsides by 6 months of treatment in some patients. Nevertheless, we demonstrate a sustained 24% shortfall to perfect health performance in patient-reported overall health scores in patients treated with givosiran over an extended period. Evidence from qualitative data offered by patient experience surveys suggests that some patients experience an overall lack of energy that felt persistent under givosiran. It is unclear if this is linked to givosiran treatment or is a consequence of their underlying disease. Further research is required to elucidate the cause of the ongoing lack of energy in almost all AIP patients undergoing givosiran therapy [43].

## 5. Conclusions

Our study shows that givosiran treatment has the potential to transform the lives of patients suffering from AIP with a range of symptoms, including those with chronic symptoms without attacks, with a generally satisfactory AE profile. Early treatment with givosiran was associated with a better clinical response. Further research is required to evaluate whether patients receiving lower or less frequent doses of givosiran are at a reduced risk of AEs whilst continuing to be protected from acute episodes [36]. This is particularly relevant for givosiran injections, where the estimated cost is >USD 400,000 per patient per year [45]. Treatment costs (e.g., hemin infusion and intensive care), however, must be considered together with the potential for improvement in patient HR-QoL and the long-term savings associated with fewer healthcare interactions.

## Figures and Tables

**Table 1:** Patient characteristics, disease profile and treatment history for AIP.

Total patient number	n = 28
Age (mean ± SD)	42.7 years (±14.8)
Sex	23f:5m
Porphyria classification	100% AIP, with heterogenous mutations
Duration between diagnosis and givosiran treatment, mean (range):	110.9 months (1–324)
Duration of givosiran treatment, mean (range):	30 months (3–67)
Classification (according to Ipnet defined terms)	
Sporadic Attacks—Group 1	n = 13 (46.4.%)
Symptomatic High Excreter—Group 2	n = 5 (17.9%)
Prophylactic Heme—Group 3	n = 5 (17.9%)
Recurrent attacks—Group 4	n = 5 (17.9%)
Treatment administered, prior to givosiran:	
Glucose	n = 28 (100%)
Heme (acute)	n = 26 (92.8%)
Heme (prophylactic)	n = 6 (21.4%)
GnRH	n = 1 (3.6%)
Opioids	n = 8 (28.6%)

**Table 2:** Side effects and adverse events experienced by AIP patients undergoing treatment with givosiran for at least 6 months (*n* = 26).

Side Effects and Adverse Events (AE)	Patients % (n = 26)
Decreased renal function	30.7 (8)
GFR < 30 mL/min	11.5 (3)
Renal failure	0
Muscular cramps	38 (10)
Nausea	27 (7)
Pancreatitis	0
Vascular events	3.8 (1)
Liver enzyme elevation < 1.5 ULN	15.4 (4)
Liver enzyme elevation > 1.5 ULN	7.7 (2)
Homocysteinemia	100 (26)

## Data Availability

The datasets presented in this article are not readily available because to the German Data Protection. Requests to access the datasets should be directed to authors. Data are contained within the article and Supplementary Materials.

## Supplementary Materials

The following supporting information can be downloaded at https://www.mdpi.com/article/10.3390/jcm13226779/s1. Supplementary File S1: EQ-5D-5L; Supplementary File S2 [46]: Fatigue Assessment Scale (FAS); Supplementary Table S1: HMBS Gene Mutations.
