# Supplementary material for: German Real-World Experience of Patients with Diverse Features of Acute Intermittent Porphyria Treated with Givosiran
Source: J Clin Med. 2024 Nov 11;13(22):6779. doi: 10.3390/jcm13226779 (PMC11594983; doi:10.3390/jcm13226779)
Supplement: Supplementary file 1 [file jcm-13-06779-s001.zip › jcm-3216289-supplementary.pdf]

Supplementary Files

German Real-World Experience of Patients with Diverse Features of Acute Intermittent Porphyria Treated with Givosiran

Ilja Kubisch 1,†, Nils Wohmann 1,†, Thaddäus Till Wissniowski 1, Thomas Stauch 2, Lucienne Oettel 1,
Eva Diehl-Wiesenecker 3, Rajan Somasundaram 3 and Ulrich Stölzel 1,*

1 Porphyria Center, Chemnitz Hospital, 09116 Chemnitz, Germany; i.kubisch@skc.de (I.K.); n.wohmann@skc.de (N.W.); t.wissniowski@skc.de (T.T.W.); l.oettel@skc.de (L.O.)
2 MVZ Labor Volkmann, 76131 Karlsruhe, Germany; t.stauch@laborvolkmann.de
3 Department of Emergency Medicine and Porphyria Clinic, Charité—Universitätsmedizin Berlin, Corporate Member of Freie Universität Berlin and Humboldt-Universität zu Berlin, 10115 Berlin, Germany; eva.diehl-wiesenecker@charite.de (E.D.-W.); rajan.somasundaram@charite.de (R.S.)
* Correspondence: dr.stoelzel@porphyrie.de
† These authors contributed equally to this work.

Supplementary File S1. EQ-5D-5L

EQ-5D-5L

Health Questionnaire

English version for the UK

Sample

UK (English) © 2009 EuroQol Group EQ-5D™ is a trade mark of the EuroQol Group

Under each heading, please tick the ONE box that best describes your health TODAY.

MOBILITY

- I have no problems in walking about ☐
- I have slight problems in walking about ☐
- I have moderate problems in walking about ☐
- I have severe problems in walking about ☐
- I am unable to walk about ☐

SELF-CARE

- I have no problems washing or dressing myself ☐
- I have slight problems washing or dressing myself ☐
- I have moderate problems washing or dressing myself ☐
- I have severe problems washing or dressing myself ☐
- I am unable to wash or dress myself ☐

USUAL ACTIVITIES *(e.g. work, study, housework, family or leisure activities)*

- I have no problems doing my usual activities ☐
- I have slight problems doing my usual activities ☐
- I have moderate problems doing my usual activities ☐
- I have severe problems doing my usual activities ☐
- I am unable to do my usual activities ☐

PAIN / DISCOMFORT

- I have no pain or discomfort ☐
- I have slight pain or discomfort ☐
- I have moderate pain or discomfort ☐
- I have severe pain or discomfort ☐
- I have extreme pain or discomfort ☐

ANXIETY / DEPRESSION

- I am not anxious or depressed ☐
- I am slightly anxious or depressed ☐
- I am moderately anxious or depressed ☐
- I am severely anxious or depressed ☐
- I am extremely anxious or depressed ☐

Sample

UK (English) © 2009 EuroQol Group EQ-5D™ is a trade mark of the EuroQol Group

- We would like to know how good or bad your health is TODAY.
- This scale is numbered from 0 to 100.
- 100 means the best health you can imagine.
  0 means the worst health you can imagine.
- Mark an X on the scale to indicate how your health is TODAY.
- Now, please write the number you marked on the scale in the box below.

YOUR HEALTH TODAY = ☐

The best health you can imagine

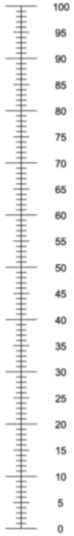

The worst health you can imagine

Sample

UK (English) © 2009 EuroQol Group EQ-5D™ is a trade mark of the EuroQol Group

Supplementary File S2.
Fatigue Assessment Scale (FAS)

Fatigue Assessment Scale (FAS)

Instructions:

The following ten statements refer to how you usually feel. Please give an answer to each question, even if you do not have any complaints at the moment.

		Never	Sometimes	Regularly	Often	Always
1	I am bothered by fatigue	**1**	**2**	**3**	**4**	**5**
2	I get tired very quickly	**1**	**2**	**3**	**4**	**5**
3	I don't do much during the day	**1**	**2**	**3**	**4**	**5**
4	I have enough energy for everyday life	**5**	**4**	**3**	**2**	**1**
5	Physically, I feel exhausted	**1**	**2**	**3**	**4**	**5**
6	I have problems to start things	**1**	**2**	**3**	**4**	**5**
7	I have problems to think clearly	**1**	**2**	**3**	**4**	**5**
8	I feel no desire to do anything	**1**	**2**	**3**	**4**	**5**
9	Mentally, I feel exhausted	**1**	**2**	**3**	**4**	**5**
10	When I am doing something, I can concentrate quite well	**5**	**4**	**3**	**2**	**1**

Developer Reference:

Michielsen, H. J., De Vries, J., & Van Heck, G. L. (2003). Psychometric qualities of a brief self-rated fatigue measure the fatigue assessment scale. Journal of Psychosomatic Research, 54, 345–352.

Supplementary Table S1. HMBS Gene Mutations

Patient No.	Sex	HMBS mutation	AIP classification (Ipnet)
1	f	Cc.440C>T, Exon 9	Prophylactic Heme
2	f	*Despite borderline low PBGD activity, no mutation found*	Prophylactic Heme
3	f	*No sequencing performed*	Sporadic
4	f	c.605dupT	Symptomatic High Exc.
5	f	c.517C>T, p.Arg173Trp heterozygot, Exon 9(10)	Recurrent
6	m	c.76C>T, p.Arg26Cys heterozygot, Exon 2	Symptomatic High Exc.
7	f	c.380delC	Sporadic
8	f	c.499C>T, p.Arg167Trp heterozygot, Exon 9(10)	Symptomatic High Exc.
9	f	c.685C-D pGlnn229*Exon 12	Sporadic
10	m	IVS1+1G>A	Sporadic
11	f	*No sequencing performed*	Prophylactic Heme
12	f	c.912-2A>G heterozygot, Exon 15	Prophylactic Heme
13	f	*No sequencing performed*	Sporadic
14	f	c380delC	Sporadic
15	m	c.331G>A, p.Gly111Arg, Exon 7	Prophylactic Heme
16	f	*No sequencing performed*	Recurrent
17	f	c833T>C p.Leu278Pro	Recurrent
18	f	*No sequencing performed*	Recurrent
19	f	*No sequencing performed*	Sporadic
20	f	*No sequencing performed*	Sporadic
21	f	c.673C>T p.(Arg225*) heterozygot, Exon 12	Sporadic
22	m	*No sequencing performed*	Symptomatic High Exc.
23	m	c.517C>T (Arg173Trp)	Sporadic
24	f	c.517C>T (Arg173Trp)	Sporadic
25	f	c.652-2delA, p.(?) heterozygot, Intron 11	Sporadic
26	f	c.500G>A, p.Arg167Gln heterozygot, Exon 9	Symptomatic High Exc.
27	f	c.730_731del p.Leu244Alafs*6	Sporadic
28	f	c.517C>T p.Arg173Trp heterozygot, Exon 9(10)	Recurrent
